# A Rare Complication of Herpes Zoster: Segmental Zoster Paresis

**DOI:** 10.1155/2016/7827140

**Published:** 2016-05-30

**Authors:** Hooi Khee Teo, Mayank Chawla, Manish Kaushik

**Affiliations:** Department of Internal Medicine, Singapore General Hospital, Singapore 169608

## Abstract

Herpes zoster is a common presentation in both the community and emergency department; however segmental zoster paresis is a rare complication that can lead to misdiagnosis. We present a case of a 74-year-old Indian gentleman with a background of well controlled diabetes mellitus, hypertension, and ischaemic heart disease who presented with sudden right lower limb weakness. This was preceded by a 5-day history of paraesthesia starting in the right foot and ascending up the right lower limb. On examination, there was a characteristic vesicular rash in the L2/3 region with MRC grading 3/5 in the right hip flexors. The rest of the neurological examination was unremarkable. MRI of the spine did not show any evidence of spinal disease. The patient was initiated on IV acyclovir with improvement of the lower limb weakness to MRC grading 5/5 as the vesicles improved. This is an interesting case as it highlights a rare presentation of zoster: segmental motor paresis that recovered fully with resolution of the rash. It shows the importance of recognizing motor neuropathy as a complication of shingles as it has a very good prognosis with most patients regaining full motor function of the affected limb with treatment.

## 1. Introduction

Herpes zoster (HZ) is caused by the reactivation of* Varicella Zoster Virus* (VZV) and commonly presents in the elderly. Approximately 50% of HZ in the United States occur in people who are 50 years or older [1] with the highest incidence occurring from the 7th decade onwards [1]. It commonly presents as dermatomal, vesicular skin lesions and is occasionally accompanied with postherpetic neuralgia. However, asymmetrical, focal motor paresis, known as segmental zoster paresis, is a rare complication [2–5]. We describe a case of segmental zoster paresis that presented acutely as loss of function of the right lower limb with the appearance of a vesicular rash occurring at the same time, and full restoration of function with resolution of the rash.

## 2. Case Report

A 74-year-old Indian gentleman with a history of diabetes mellitus, hypertension, and ischaemic heart disease, presented to the emergency department (ED) of Singapore General Hospital with sudden onset weakness of the right lower limb first noticed when he was unable to get off the toilet seat. Prior to this ED visit, the patient had presented to the same ED the day before, with a five-day history of right thigh paraesthesia starting in the right foot and gradually spreading to involve the entire right lower limb. There was no associated loss of consciousness, weakness of upper limbs, bowel or bladder symptoms, or backache. He denied history of any recent gastrointestinal or respiratory infections as well.

On examination, patient was afebrile, with stable vital signs. Of note, there was a characteristic vesicular rash on the right thigh in the L2/3 region and isolated vesicles in other dermatomes, which were not apparent on the first ED visit. Examination of lower limbs showed reduced power in right hip flexors (MRC grading 3/5). The power was MRC 5/5 in all other muscle groups of the right lower limb and in all muscle groups of the left lower limb. There was hyperaesthesia noted over the right thigh in the L2/3 dermatome. The deep tendon reflexes were normal in both the upper and lower limbs. In addition, examination of the cranial nerves was normal and plantar reflex was downgoing bilaterally. The anal tone was intact.

A diagnosis of multidermatomal HZ infection was made: more than 20 lesions were noted in different dermatomes. The patient was admitted and commenced on intravenous acyclovir and symptomatic treatment. He was also investigated for sudden onset of weakness of the right lower limb. Magnetic Resonance Imaging (MRI) of the thoracic and lumbar spine performed revealed no evidence of spinal disease other than a focal posterior annular tear at T12/L1 disc. The orthopaedic service was consulted, and they opined that the above was an incidental MRI finding and was not accountable for the sudden right lower limb weakness. No nerve conduction studies were done. During the admission, as the vesicular rash resolved, the weakness in the right lower limb improved significantly. By day 7, patient was able to bear weight and, subsequently, to mobilize independently by discharge. However, he did continue to have residual postherpetic neuropathic pain.

## 3. Discussion

This case is interesting as it highlights one common and one rare aspect of HZ, respectively: (1) the often described prior ED attendance for nonspecific symptoms before appearance of the characteristic vesicular rash and (2) segmental motor paresis that started with appearance of the rash and recovered fully with resolution of the rash.

It is not uncommon for patients to describe nonspecific flu-like symptoms or tingling sensations, prior to the onset of the characteristic clustered vesicular rash of HZ. As such, many of these patients are only diagnosed in retrospect, when the rash manifests. In order to allow early diagnosis and consequent reduction in postherpetic pain, physicians should have a high suspicion for HZ especially in high-risk patients: the elderly and immunocompromised individuals.

Diabetes mellitus (DM) is a common chronic condition managed in the community. In 1983, Ragozzino et al. did a study of residents in Rochester, Minnesota, and concluded that DM was not a risk factor for developing HZ [6]. However, in recent years, more publications have revealed that patients with a background of DM are at risk of developing HZ [7]. In fact, there was 1.8- to 8.4-fold increase in risk of developing herpes zoster [8] in diabetics at Osaka Kitano Hospital and, in a large study in Spain, an adjusted relative risk of 3.7 (95% CI 2.0–6.8) in the 30–44-year old group [7]. Reasons as to why this occurs in DM are still debatable, but some claim that it could be due to changes in the immune system of diabetics [6]. With reports of increased risk of developing HZ in diabetic individuals, physicians would need to have a high level of suspicion of HZ in diabetics presenting with nonspecific symptoms as in this patient.

Segmental zoster paresis (SZP) is a rare complication in herpes zoster infection [3, 5] and most authors report it as occurring a few days to weeks after the appearance of the herpetic rash [2, 3]. It is more common in the elderly and weakness often occurs in the proximal muscle groups (C5–7, L2–4), as with this patient [9–12]. SZP is better known in the medical fraternity as Bell's palsy, resulting from herpes zoster affecting the facial nerve, accounting for close to 50% of reported SZP cases [2]. There have, however, been exceptions with reports of SZP occurring prior to the rash [4] or affecting distal muscle groups [3].

A literature search showed that patients with paresis or radiculopathy resulting from HZ often take weeks to months before full return of function of the affected limb, long after the rash has resolved [13, 14]. This patient however had full return of function by day 8 of admission, as the vesicles started to crust.

It is known that, with previous varicella infection, the virus lies dormant in the dorsal root ganglia and, on reactivation, results in the commonly described sensory symptoms of hyperesthesia or paraesthesia in the affected dermatome [1]. However, the pathogenesis behind development of SZP is still poorly understood [2]. Some postulate that local inflammation around the dorsal root ganglion causes hypervascularity in the perineural structure or disruption of the blood nerve barrier, resulting in motor deficit [8, 15]. Another popular theory is that the spread of the varicella virus to the anterior horn cells results in paresis or radiculopathy if spread is to the anterior spinal nerve roots [2].

## 4. Conclusion

Herpes zoster viral infection is commonly seen in the community, but motor neuropathy is rarely seen and is often treated separately from the vesicular rash or as being secondary to an independent spinal pathology. It is important to recognize motor neuropathy as a complication of shingles and treat it promptly as it has a very good prognosis [2], with most patients regaining full motor function of the affected limb with treatment of the underlying infection.
